# The regional impact of an epidemic: socioeconomic and demographic data in Java, 1905-1924

**DOI:** 10.1016/j.dib.2021.107710

**Published:** 2021-12-13

**Authors:** Daniel Gallardo-Albarrán, Pim de Zwart

**Affiliations:** Wageningen University, The Netherlands

**Keywords:** Southeast Asia, Java, Indonesia, Sugar Industry, Globalization, 1918 Influenza Pandemic, Mortality, Wages

## Abstract

This dataset contains data for the island of Java, Indonesia, at the *regency*-level – comparable to present-day *kabupaten*. The data concern trends in area of cultivated sugar, total and per-hectare sugar production, crude mortality rates and wages in the period ca. 1909-1924. In addition to this panel dataset, cross-sectional figures were collected about the amount of sawah land (1920), urbanization rates (1905), medical personnel (1919), native population (1905) and areas with communal property with rotating shares. These figures were gathered from primary documents published by the Dutch colonial government and its constituent agencies. These data are relevant for all social scientists (such as economists, demographers or economic historians) interested in Southeast Asia or in the relationship between health indicators and economic development before, during and after an unprecedented pandemic. Historians of Southeast Asia and Indonesia may be interested in these figures as a background against which developments in politics and culture may be sketched. In addition, epidemiologists assessing the health consequences of the 1918 influenza pandemic will find valuable information in this regional dataset. Gallardo-Albarrán and de Zwart (2021) have shown on the basis of this dataset how the 1918 influenza pandemic affected economic activity across Java in this period.

## Specifications Table

**Table:** 

Subject	Economics, History
Specific subject area	Economic history, Demographic history, Colonial history, Asian history
Type of data	Cross-sectional and panel data
How data were acquired	Data were gathered from primary documents published by the Dutch colonial government in the East Indies (present-day Indonesia).
Data format	Stata and CSV files. Filtered data. We processed and aggregated the raw data series to be able to present a consistent series of figures at the administrative level of the *regency* as discussed in the “Experimental Design, Materials and Methods” section below*.*
Parameters for data collection	Data on relevant socioeconomic and demographic indicators that were available at the regency-level (administrative level that is comparable with present-day *kabupaten*) for Java, Indonesia. The selection of data was based on availability. We collected *all* indicators of social-economic progress in this period that were published for the *regency level* by different colonial offices in the period 1905-1924. Period (early twentieth century) was chosen to allow an analysis of the impact of the influenza pandemic of 1918. Precise beginning (1905) and ending (1924) years were determined by data availability.
Description of data collection	Data entry on the basis of primary printed documents.
Data source location	Primary historical documents were used in data collection: Koloniale Verslagen Mededeelingen Burgerlijke Geneeschkundige Dienst Onderzoek Mindere Welvaart Landbouwatlas These documents were found in the academic libraries of Utrecht University, Leiden University and Wageningen University in the Netherlands.
Data accessibility	With the article
Related research article	D. Gallardo-Albarrán, P. de Zwart, A bitter epidemic: the impact of the 1918 influenza on sugar production in Java, *Economics & Human Biology* (2021). https://doi.org/10.1016/j.ehb.2021.101011

## Value of the Data

• This dataset presents unique and highly detailed, regionally disaggregated, insights on various aspects – export production, demography and labour markets - of a colonial developing economy in the early twentieth century before, during and after the unprecedented 1918 influenza pandemic.
• These data are relevant for all social scientists (such as economists, demographers or economic historians) interested in the economic and demographic history of developing countries. The figures are particularly valuable for all historians working on Southeast Asia, and Indonesia in particular.
• These data may be used to investigate different hypotheses regarding the relationship between the colonial sugar export industry and a host of economic and demographic variables.
• The figures serve as important background information against which political and cultural developments in early-twentieth century Java may be understood.
• Epidemiologists and demographers will find value in this dataset that allows assessing the magnitude of the 1918 influenza pandemic with highly disaggregated crude mortality rates.

## 1 Data Description

Table 1 provides the summary statistics and additional details of the variables collected. The data were available for the various regencies of Java. The “regency” is a level of the colonial administration below that of the Residency and above the district. Our data also indicates in which Residency a regency was located: the Residency was the highest administrative unit of the Dutch East Indies. Early twentieth-century regencies are comparable to present-day *kabupaten* in Indonesia. We differentiate between ‘panel data variables’ (i.e. with regency-level information for various years during the period 1909-1924) and ‘cross-sectional variables’ (i.e. with regency-level information in a single year).^1^ In brackets, we specify the measurement units of each variable. The values for this table were obtained in Stata using the file ‘Table1.do’ and its associated data files (panel.dta and and cross_section.dta).

**Table 1:** Panel data and cross-sectional variables on Java's socioeconomic and demographic characteristics.

	Abbreviation in Dataset	Observations	mean	st. dev.	min.	max.
*Panel Data Variables*						
Total sugar production (thousand picul.)	sugar_prod	627	5757	5100.567	431.538	26271.3
Per-hectare sugar production (picul per hectare)	sugar_prod_area	622	1683.289	259.076	759.75	2861.394
Mortality, 1912-1924 (per 1,000 people)	mortality	1042	22.846	9.599	10.000	89.000
Wages agricultural workers, 1915-1919 (1917=100)	wage_agriculture	361	102.201	15.808	52.414	195.180
Wages plantation workers, 1915-1919 (1917=100)	wage_plant	384	103.071	21.258	31.623	223.607
*Cross-sectional variables*						
Crude Death Rates in 1918 (per 1,000 people)	mort1918	81	38.704	14.477	14	89
Crude Death Rates in 1919 (per 1,000 people)	mort1919	81	30.889	13.581	14	84
Sawah area in 1920 (total hectares)	sawah_hect_1920	79	39522.72	22793.64	6255.1	133980.5
Sawah area in 1920 (hectares per capita)	sawah_hect_1920_pc	79	0.091	0.037	0.025	0.240
Medical personnel in 1919 (per 1,000 people)	total_medical_1919_pc	80	0.012	0.005	0.003	0.033
Native population in 1905 (in thousands)	native_population1905	81	362.256	151.616	99.32	819.326
Urbanization in 1905 (in percentages)	urb_share3000	79	5.652	6.545	0	42.958
Rotating shares areas in 1903 (1 if present, 0 if not)	rotating_shares_1903	82	0.402	0.493	0	1

**Fig. 1:**
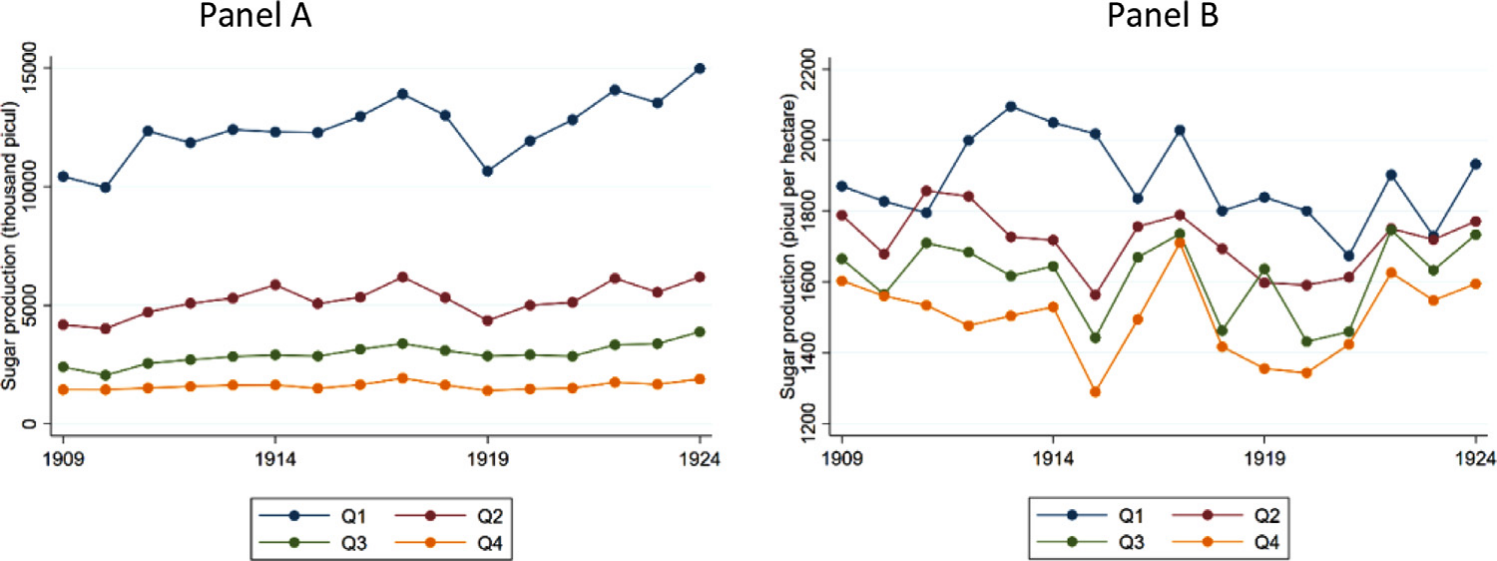
Total and per-hectare sugar production in Java, 1909-1924.

Fig. 1 shows the development of total and per-hectare sugar production across different parts of Java in the period 1909-1924, in Panel A and B, respectively. To show the degree of regional and temporal variation, the regencies were grouped into 4 categories according to their average total and per-hectare production during the analysed period. Beginning with Panel A, the series *Q1* refers to regencies in the first quartile of average sugar production between 1909 and 1924, *Q2* shows data for regencies in the second quartile, and *Q3* and *Q4* refer to the third and fourth quartile, respectively. Concerning Panel B, the series *Q1* shows average sugar production per hectare for regencies in the first quartile of average sugar production per-hectare between 1909 and 1924, *Q2* refers to the second quartile, etcetera. These figures were obtained in Stata using the file ‘Figure1.do’.

**Fig. 2:**
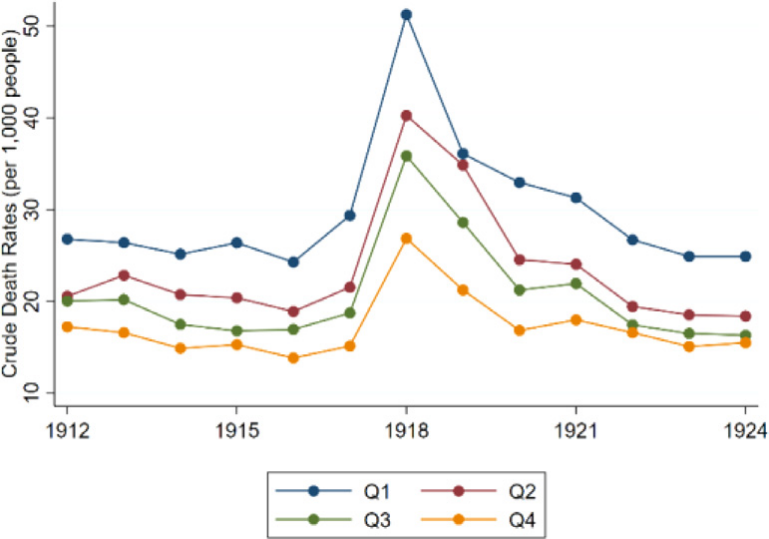
Crude death rates in Java, 1912-1924.

Fig. 2 presents trends in crude mortality rates across different regencies of Java in the period 1912-1924. Similar to Fig. 1, we show four series to appreciate health developments in the island both across space and time. We grouped the regencies into 4 categories according to the average level of mortality during the considered period. *Q1* refers to regencies in the first quartile of average mortality during the period 1912-1924, and the series *Q2, Q3* and *Q4* present information for the remaining quartiles. This figure was obtained in Stata using the file ‘Figure2.do’.

**Fig. 3:**
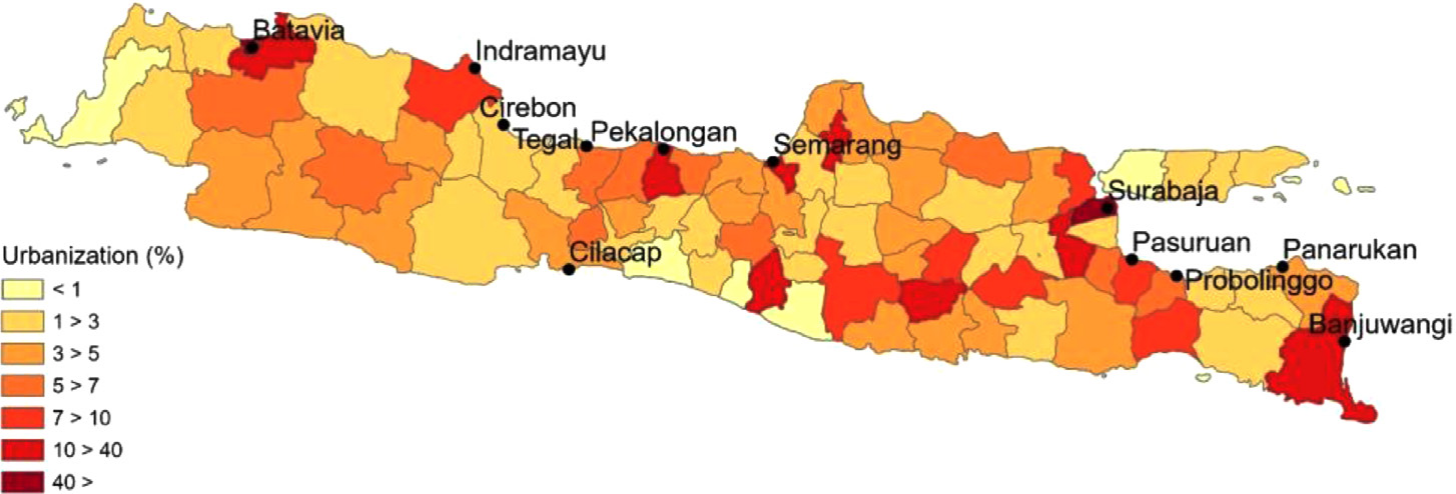
Urbanization rate in the various regencies of colonial Java, 1905. Sources: see text.

In Fig. 3, the spatial distribution of the urbanization rates in 1905 across Java are shown, measured as the percentage of people in a regency that live in a town with over 3,000 inhabitants. For this visualization, seven classes were defined that contain roughly similar numbers of regencies. First are those regencies with less than 1% urban population. This concerns, for example, the regency of Pandeglang in the south west of Java. Then there are many regencies across the island that have an urbanization rate of somewhere between 1 and 10%. In 8 regencies that had comparatively large cities, such as Pekalongan, Semarang and Banyuwangi, urbanization was between 10 and 40%.

**Fig. 4:**
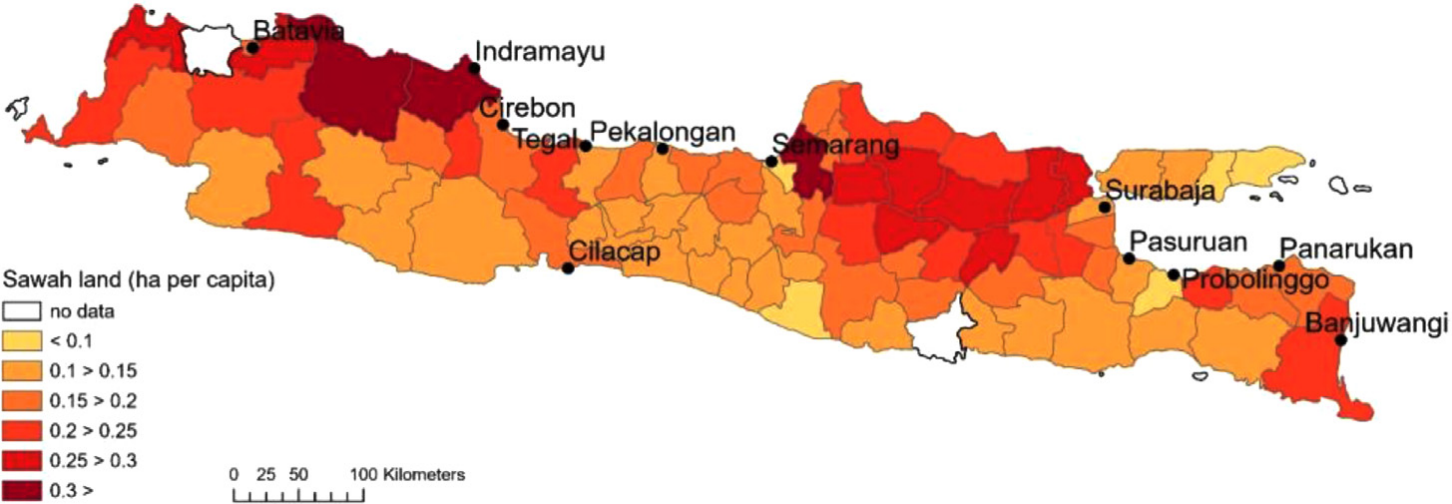
Hectare of sawah (irrigated rice field) per capita, 1920. Sources: see text.

Fig. 4 shows the amount of *sawah* (irrigated rice field) in hectare per capita. This indicator contains information about the agricultural capacity of a region relative to the total number of people. The visualization shows 7 categories of data, but one of those concerns missing observations. The amount of sawah for the regency of Tangerang (west of Batavia) and Trenggalek (central southern part of the island) were unknown. Besides this there were areas with less than 0.1 ha of *sawah* per capita. In general, the amount of *sawah* per capita seems to be somewhat higher in the northern parts of the islands (which were generally lower lying areas) compared with the southern regencies of the island where more mountains were located. Both Figs. 3 and 4 were created exploiting cross_section.dta and using ArcGIS Pro software.

## 2 Experimental Design, Materials and Methods

Data were collected from a variety of primary documents published by the Dutch East Indies colonial government and its agencies: *Mededeelingen Burgellijke Geneeskundige Dienst* (1923-1927), *Koloniaal Verslag* (1907-1925) and *Landbouwatlas* (1922).

We first consulted the Annual Reports of the Civil Medical Service (*Mededeelingen Burgerlijke Geneeskundige Dienst, MBGD*) – published between 1923 and 1927 – to obtain crude mortality rate for 81 regencies in Java for the period 1912-1927 [1]. This source has some limitations since the quality of registration of births and deaths was far from perfect. According to Chandra [2], the reported figures in these reports are likely underestimates of the true crude death rates. His estimates of the mortality impact of the influenza pandemic suggest that death rates may have actually been 4 times higher than those reported by the Civil Medical Service. Other studies in the field, however, suggest that the undercounting was much less severe than implied by Chandra [3,4]. Indeed, one can argue that the Civil Medical Service trusted its mortality numbers since they published them, whereas the same was not true for birth rates as they considered them inaccurate [5]. While we acknowledge the existence of different views about the mortality data, we have not made any adjustments due to the lack of consensus on the extent of underreporting. Also, this is not the focus of our paper, since we are interested in (1) trends in mortality over time and (2) regional differences in the mortality. We argue that potential reporting issues do not affect in a systematic (i.e. non-random) way spatial and temporal mortality patterns.

The second phase of our data collection process focused on obtaining information about sugar production. For this purpose, we used the information contained in the *Koloniaal Verslag* (Colonial Report, or KV, editions between 1911-1925) [6]. This source allowed us to create a dataset with annual information on sugar production and sugar area in use during the time period 1909-1924. Our data is so detailed and disaggregated that we considered the returns of 220 separate sugar factories that was then aggregated to match the regency-level mortality figures. As we did with the mortality data, we checked the validity and reliability of the Colonial Reports. These reports published information on sugar area and production that was sent to the government on an annual basis by private factories. There is evidence that the published information was very accurate, as one contemporary source indicates [7]. In order to provide a reliable series, we had to harmonize the data. The Colonial Reports indicate when an estate did not produce any sugar (zero production) in a given year, or whether the factory had failed to return figures to the government (missing data). In a few instances, we encountered unusual cases in which a specific estate suddenly disappeared from the reports. We think this could be due to the closing of a factory (i.e. zero production) or failure to report to the government (i.e. missing data). We dealt with this conservatively and treated these instances as missing production data. Another challenge during the data harmonization process was that data for some of the factories in a regency were missing for a few years, which we also dealt with in a conservative manner. More specifically, we set to missing data for a regency in a given year, if production numbers were missing for one or two factories in a given regency during only one or two years. If, on the other hand, production numbers were missing for one factory for more than 2 years, and more than two factories were in operation in the regency, we omitted the information for that particular factory, while data for the remaining factories were aggregated to get regency-level production figures.

For four distinct reasons, we argue that these figures are reliable and provide representative information on the sugar industry and its economic performance during the period 1905-1929. First, the same sources have been used in other research on sugar production and productivity in Java. Previous studies only analyzed these data at the aggregate level for Java as a whole, but the same data underlies it and are deemed reliable by the authors of those studies [8]. Second, we checked the main trends we obtain from our disaggregated figures with those reported in other literature that analyzed aggregated trends [9], and found they matched, giving additional confidence in the results. Third, there were no changes in the borders of the regencies in the period we analyze that could influence the time-series of sugar and mortality data. Fourth, we compared the spatial distribution of the sugar production data from the *Koloniaal Verslag* with figures on regency-level refined sugar output from the Landbouwatlas in 1922 [10]. The data from the two seperate sources show virtually identical spatial patterns as the correlation coefficient is 0.96. Thus, we regard this information as accurate of sugar output and differences across regencies.

In addition, as we, in accompanying work [11], were interested in the relationship between the influenza mortality shock and wider economic effects, we also collected data on the wages of labourers on plantations and those of labourers working in local rice agriculture. By the early twentieth century, Java had become a labour abundant island and a large share of the total population were dependant on wages as their main source of income. Wage data were collected for the regency level for the years 1914-1919 from the *Koloniale Verslagen*. These detailed series of wage data – unique for any colonial society – unfortunately were no longer collected and printed in the KV after 1919. One issue with the wages reported by the KVs is that they give minimum and maximum rates, rather than median or average wages [12]. For some years and some regencies, the difference between the two rates could be considerable: a plantation coolie wage in Surakarta in 1915, for example, could range between 0.20 and 0.50 *fl.* per day. Such large ranges were the exception, however, and more often the range would be much smaller. To calculate an average wage on the basis of these rates, we follow the generally accepted approach in historical wages literature of taking the log-normal average between the two values as it is assumed that the lower wages were paid more often than the higher rates. This also fits with evidence from other contemporary reports that suggest the majority of workers earned wages that were nearer the lower end of the scale [12].

Finally, we assembled figures cross sectional figures on economic and medical contextual indicators. Data on urbanization rate in 1905 were taken from the population count that was published in the *Koloniaal Verslag* of 1907 [6]. According to Jacob van Gelderen, the director of the Dutch East Indies Statistical Office (*Centraal Kantoor voor de Statistiek*) between 1925 and 1932, this population count, and the ones preceding it from the late 19^th^ century were actually reasonably accurate with regard to the figures for Java (but not so those of the other islands of the Dutch East Indies) [13]. Data on the amount of *sawah* per person in 1920 were taken from the *Landbouwatlas* [10]. The *Landbouwatlas* was published by the Statistical Office on the basis of the annual and monthly reports of local officials who kept detailed records of local harvests for tax purposes. The Statistical Office itself assessed the figures as “sufficiently accurate” [10]. Cross-sectional indicators on the provision of health care across the island in 1919 were obtained from the “Brief report” of the Civil Medical Service (BGD) [5]. In this report, the BGD provides an overview of its activities across the island over the years 1912 to 1919. Among the data listed in these figures are the numbers of different types of medical personnel and outposts, e.g. dentists, vaccine doctors, apothecaries, and the number of stations of the BGD and other (western) health care providers. These figures are assumed to be accurate as the BGD would have had a good image of its personnel and their locations.

## Ethics Statement

These data were collected without the involvement of human subjects, they were not gathered via animal experiments or social media platforms. No ethical issues are related to these figures.

## CRediT Author Statement

**Daniel Gallardo-Albarrán:** Conceptualization, Methodology, Software, Validation, Formal Analysis, Data Curation, Writing, Visualization (Graphs); **Pim de Zwart:** Conceptualization, Methodology, Software, Investigation, Resources, Data Curation, Writing, Visualization (Maps).

## Declaration of Competing Interest

The authors acknowledge financial support from the Dutch Research Council (NWO) for funding provided for the projects “Unfair Trade: Globalization, Institutions and Inequality in Southeast Asia, 1830-1940” (NWO Veni 275-53-016) and “Global health inequality and the diffusion of sanitation since 1850” (NWO Veni 201H.048). The authors declare that they have no known competing financial interests or personal relationships which have or could be perceived to have influenced the work reported in this article.

## Appendix Supplementary materials

Image, application 1

## Footnotes

1 For the variable urbanization rates, we omitted *Batavia* from the sample, as we calculated urbanization rates by dividing the number of city dwellers over the total native population. This leads to a slight upward bias in urbanization rates in general, but in Batavia in particular because there were so many Europeans and Chinese living there: this resulted in an urbanization rate there of 139 percent.
